# Whole Genome Analysis of African G12P[6] and G12P[8] Rotaviruses Provides Evidence of Porcine-Human Reassortment at NSP2, NSP3, and NSP4

**DOI:** 10.3389/fmicb.2020.604444

**Published:** 2021-01-12

**Authors:** Fortunate Mokoena, Mathew Dioh Esona, Luyanda Mapaseka Seheri, Martin Munene Nyaga, Nonkululelo Bonakele Magagula, Arnold Mukaratirwa, Augustine Mulindwa, Almaz Abebe, Angeline Boula, Enyonam Tsolenyanu, Julia Simwaka, Kebareng Giliking Rakau, Ina Peenze, Jason Mathiu Mwenda, Maphahlaganye Jeffrey Mphahlele, Andrew Duncan Steele, African Rotavirus Surveillance Network

**Affiliations:** ^1^Department of Biochemistry, Faculty of Natural and Agricultural Science, North West University, Mmabatho, South Africa; ^2^Diarrhoeal Pathogens Research Unit, Department of Virology, Sefako Makgatho Health Sciences University, Pretoria, South Africa; ^3^Next Generation Sequencing Unit and Division of Virology, Faculty of Health Sciences, University of the Free State, Bloemfontein, South Africa; ^4^Department of Medical Microbiology, University of Zimbabwe-College of Health Sciences, Harare, Zimbabwe; ^5^Mulago National Referral Hospital, Kampala, Uganda; ^6^Ethiopian Public Health Institute, Addis Ababa, Ethiopia; ^7^Mother and Child Center, Chantal Biya Foundation, Yaoundé, Cameroon; ^8^Department of Paediatrics, Sylvanus Olympio Teaching Hospital of Lome, Lome, Togo; ^9^Virology Laboratory, University Teaching Hospital, Lusaka, Zambia; ^10^African Rotavirus Surveillance Network, Immunization, Vaccines and Development Cluster, WHO African Regional Office, Brazzaville, Congo; ^11^Enteric and Diarrheal Diseases, Global Health, Bill & Melinda Gates Foundation, Seattle, WA, United States

**Keywords:** Africa, whole genome sequencing, next generation sequencing, group A rotaviruses, genotype 12, reassortment

## Abstract

Group A rotaviruses (RVA) represent the most common cause of pediatric gastroenteritis in children <5 years, worldwide. There has been an increase in global detection and reported cases of acute gastroenteritis caused by RVA genotype G12 strains, particularly in Africa. This study sought to characterize the genomic relationship between African G12 strains and determine the possible origin of these strains. Whole genome sequencing of 34 RVA G12P[6] and G12P[8] strains detected from the continent including southern (South Africa, Zambia, Zimbabwe), eastern (Ethiopia, Uganda), central (Cameroon), and western (Togo) African regions, were sequenced using the Ion Torrent PGM method. The majority of the strains possessed a Wa-like backbone with consensus genotype constellation of G12-P[6]/P[8]-I1-R1-C1-M1-A1-N1-T1-E1-H1, while a single strain from Ethiopia displayed a DS-1-like genetic constellation of G12-P[6]-I2-R2-C2-M2-A2-N2-T2-E2-H2. In addition, three Ethiopian and one South African strains exhibited a genotype 2 reassortment of the NSP3 gene, with genetic constellation of G12-P[8]-I1-R1-C1-M1-A1-N1-T2-E1-H1. Overall, 10 gene segments (VP1–VP4, VP6, and NSP1–NSP5) of African G12 strains were determined to be genetically related to cognate gene sequences from globally circulating human Wa-like G12, G9, and G1 strains with nucleotide (amino acid) identities in the range of 94.1–99.9% (96.5–100%), 88.5–98.5% (93–99.1%), and 89.8–99.0% (88.7–100%), respectively. Phylogenetic analysis showed that the Ethiopian G12P[6] possessing a DS-1-like backbone consistently clustered with G2P[4] strains from Senegal and G3P[6] from Ethiopia with the VP1, VP2, VP6, and NSP1–NSP4 genes. Notably, the NSP2, NSP3, and NSP4 of most of the study strains exhibited the closest relationship with porcine strains suggesting the occurrence of reassortment between human and porcine strains. Our results add to the understanding of potential roles that interspecies transmission play in generating human rotavirus diversity through reassortment events and provide insights into the evolutionary dynamics of G12 strains spreading across selected sub-Saharan Africa regions.

## Introduction

Acute gastroenteritis is the fourth leading cause of mortality in infants and young children <5 years old worldwide (Liu et al., 2016). Group A rotaviruses (RVA), primarily associated with an estimated 128 500 deaths per annum, are the most important virus induced infectious agent of gastroenteritis in children <5 years old and Africa and Asia bear the most burden of RVA disease (Troeger et al., 2018).

The rotavirus genus belongs to the *Reoviridae* family and is composed of 11 segments of double stranded RNA (dsRNA) encoding six structural (VP1-VP4, VP6 and VP7) and six non-structural proteins (NSP1-NSP6), which are enclosed in a triple-layered capsid. The two outer capsid proteins, VP4 (Protease-sensitive protein) and VP7 (Glycoprotein), are important for inducing neutralizing antibodies and protective immunity in the host. Based on the genetic diversity of VP4 and VP7, a widely used binary classification system in RVA surveillance studies is in place as G-type and P-type, respectively (Estes and Greenberg, 2013). The traditional binary classification system has been extended to assign genotypes to all 11 genes (Matthijnssens et al., 2008). Based on the whole genome analyses, most human RVAs show high sequence similarity in all their genes to either the Wa-like (I1-R1-C1-M1-A1-N1-T1-E1-H1) or DS-1-like (I2-R2-C2-M2-A2-N2-T2-E2-H2) prototype strains and to a lesser extent to the AU-1-like strains, which are characterized by I3-R3-C3-M3-A3-N3-T3-E3-H3 (Matthijnssens et al., 2008; Matthijnssens and Van Ranst, 2012). These two classification systems have accelerated the understanding of the genetic diversity of RVA. As of May 2018, human and animal RVA have been classified into 36G and 51P genotypes^1^. Globally, genotypes G1P[8], G2P[4], G3P[8], G4P[8], G9P[8], and G12P[8] are the most commonly identified cause of human infections (Santos and Hoshino, 2005; Doro et al., 2014; Esona and Gautam, 2015).

In the last decade, novel genotypes such as G12P[6] and G12P[8] have gained epidemiological relevance as frequently detected RVA globally and in lower-middle-income countries (Page et al., 2009; Bányai et al., 2012; Doro et al., 2014; Seheri et al., 2014;2017). Genotype G12 was first identified in children with acute diarrhea in the Philippines in 1987 (Taniguchi et al., 1990; Urasawa et al., 1990). A decade after the first identification, G12 strains were reported more frequently globally, in association with the VP4 P[6] or P[8] genotype, and sporadically, with P[4] and P[9] (Pongsuwanna et al., 2002; Wakuda et al., 2003; Samajdar et al., 2006; Rahman et al., 2007; Matthijnssens et al., 2009, 2010; Mwenda et al., 2010). The African continent has reported an increased prevalence of G12 strains since 2009 (Mwenda et al., 2010; Seheri et al., 2017). G12 strains were first reported in Malawi and South Africa in diarrheic stools of children (Page et al., 2009). Interestingly, most of these early strains were associated with VP4 P[6], a traditionally animal genotype, and it was thus proposed that this might be a mechanism whereby a novel genotype might intrude into human populations to adapt for biological competitiveness (Cunliffe et al., 2009; Page et al., 2009). This was followed by reports of G12 strains circulating as prominent genotypes in multiple African countries, such as Malawi (Nakagomi et al., 2012), Cameroon (Ndze et al., 2013a), Democratic Republic of the Congo (Pukuta et al., 2014), Uganda (Odiit et al., 2014), Senegal (Dia et al., 2015), Tunisia (Moussa et al., 2017), Mozambique (Joao et al., 2018), Central African Republic (Moure et al., 2018), Nigeria (Japhet et al., 2018), and Ethiopia (Abebe et al., 2018), over recent years. Although less common, animal G12 strains have also been detected in porcine and bovine from Cameroon (Ndze et al., 2013a) and Uganda (Bwogi et al., 2017) and South Africa (unpublished data from this laboratory).

The G12 strains are now considered common human rotavirus strains, circulating worldwide (Matthijnssens et al., 2011). Though, the precise origin remains elusive, it is suspected that porcine and bovine species are the natural reservoir hosts of this rotavirus VP7 genotype (Rahman et al., 2007; Midgley et al., 2012; Ndze et al., 2013a). Another hypothesis suggests that G12 strains may have originated from an unusual genomic plasticity, driven by multiple reassortment events (Rahman et al., 2007). In this regard, a better understanding of the global emergence and dissemination of G12 strains requires information about the whole genome characterization of these RVA strains.

The whole genome classification of RVA enabled endeavors aimed at deciphering evolutionary relationships between human and animal strains (Ghosh and Kobayashi, 2011; Matthijnssens et al., 2011). As of 2020 only a few African human G12 strains had undergone whole genome characterization, including G12P[4] (strain 3133WC) and G12P[6] (3176WC) from South Africa (Jere et al., 2011), G12P[6] (KDH633 and KDH684) and G12P[8] (KDH651) from Kenya (Komoto et al., 2014), six Malawian strains, of both G12P[6] (MAL39, MAL40, and MAL88) and G12P[8] (MAL38, MAL65, and MAL66) (Nakagomi et al., 2017) and five similar Mozambican strains, G12P[6] (0042, 0050, 0278, and 0289) and G12P[8] (0060a) (Strydom et al., 2019).

Given the high predominance and diversity of G12 strains in Africa, we have attempted to gain insight into the degree of genetic variability, potential origin and evidence of interspecies transmission of 34 G12 strains circulating in the southern, central, eastern and western parts of Africa during the period 2004–2012 that were available in this laboratory. Sequence determination and phylogenetic analysis of all 11 genome segments from Cameroon, Ethiopia, South Africa, Togo, Uganda, Zambia, and Zimbabwe were constructed for inferring genetic relationship between human G12 strains from Africa and other circulating global G12 RVA strains.

## Materials and Methods

### Ethics Statement

The study was approved by Sefako Makgatho Health Science University (SMU) Research Ethics Committee (MREC/P/108/2013:PG). The diarrheal stool samples were collected as a routine diagnostic clinical specimen when the parents brought their child to a health facility for clinical management, requiring no written informed consent. As part of the WHO-coordinated rotavirus surveillance network, the archived rotavirus-positive specimens, were anonymized and utilized for strain characterization under a Technical Service agreement and a Materials Transfer Agreement to the WHO AFRO Regional Reference Laboratory based at Sefako Makgatho Health Services University. The WHO Research Ethics Review Committee granted an exemption activity, noting that the procedures involved in the study are part of routine hospital-based rotavirus surveillance.

### Sample Collection and Inclusion Criteria

Stool samples from several African countries were collected from hospitalized children under the age of 5 years presenting with acute gastroenteritis and stored at −20°C in sentinel or national laboratories through the African Rotavirus Surveillance Network (ARSN) coordinated by WHO AFRO (Mwenda et al., 2010). The stool samples were initially screened for RVA antigen using a commercially available enzyme immunoassay rotavirus kit (ProSpecT Rotavirus Microplate Kit, United Kingdom) in the country of origin, and positive samples were then transferred to the WHO/AFRO Regional Rotavirus Reference Laboratory (RRL) based at the Diarrhoeal Pathogens Research Unit (DPRU), Sefako Makgatho University (SMU) in Pretoria, South Africa for rotavirus molecular characterization. This study characterized the full genomes of 34 rotavirus G12 genotypes collected between 2004 and 2012 from Cameroon, Ethiopia, South Africa, Togo, Uganda, Zambia, and Zimbabwe.

### RNA Extraction and Genome Sequencing

Total dsRNA from the stool sample was extracted using established methods (Nyaga et al., 2013, 2015). For whole genome sequencing, segment-specific primers were used to amplify each rotavirus gene (Nyaga et al., 2014; Magagula et al., 2015). The PCR products were quantified using a SYBR green dsDNA detection assay (Thermo Fisher Scientific, United States). All 11 amplicons per genomic segment were pooled in equimolar amounts, sheared for 15 min and ligated with Ion Torrent compatible barcoded adapters using the Ion Xpress Plus Fragment Library Kit (Life Technologies, United States) to create 200-base pair (bp) libraries. Barcoded libraries were pooled in equal volumes and cleaned with AMPure XP Reagent (Agencourt, Bioscience, United States). Quantitative PCR was used to assess the quality of barcoded libraries and to determine the template dilution factor for emulsion PCR. The pool was diluted appropriately and amplified on Ion Sphere Particles (ISPs) on the Ion One Touch instrument (Life Technologies). The amplified pool was cleaned and enriched for template-positive ISPs on the Ion One Touch ES instrument (Life Technologies). Sequencing was performed on the Ion Torrent PGM using an Ion 316 chip. The raw sequencing reads were de-multiplexed by barcode, trimmed and *de novo* assembled using CLC Bio’s clc_novo_assemble program (Qiagen, Germany). Contigs were searched against full-length RVA segment on the non-redundant NCBI nucleotide database to find the closest reference sequence for each segment. The sequences were deposited in the NCBI GenBank, under accession numbers listed in Supplementary Table S1.

### Computational Analyses

The genotype of each of the 11 gene segments of the 34 RVA study genomes was determined using the RotaC v2.0 webserver according to the Rotavirus Classification Working Group (RCWG) guidelines (Maes et al., 2009) and the Basic Local Alignment Search Tool (BLAST) program at the National Center for Biotechnology Information website (available at: http://www.bcbi.nlm.gov/BLAST/). Maximum likelihood phylogenetic tree in Molecular Evolutionary Genetics Analysis (MEGA) vs. 6 (Tamura et al., 2013). Using corrected Akaike Information Criterion (AICc), the following substitution models were implemented GTR + G + I (VP1, VP2, VP4, VP6, VP7, NSP1 and NSP5), and HKY + G + I (VP3, NSP2, NSP3 and NSP4). The reliability of the branching order was estimated from 1,000 bootstrap replicates. For sequence comparisons, pairwise nucleotide and amino acid sequence identity matrix were generated using MEGA 6 (Tamura et al., 2013).

## Results

### Whole Genome Genotyping and Sequence Analysis of G12 Strains From Africa

The demographic characteristics of the African study strains can be found in Table 1, indicating that the data analyzed in the study represents African infants and children aged 0–32 months, the majority of whom were males. On whole genomic analysis, 13 of 14 African G12P[6] study strains displayed a Wa-like (genotype 1) constellation, with the exception of the Ethiopian strain (MRC-DPRU861), which exhibited a DS-1-like (genotype 2) genetic backbone (Table 2).

**TABLE 1:** Demographic characteristics of the G12P[6] and G12P[8] strains detected from seven African countries (CMR, Cameroon; ETH, Ethiopia; UGA, Uganda; ZAF, South Africa; TGO, Togo; ZMB, Zambia; ZWE, Zimbabwe) from 2004 to 2012.

Strain	Genotype	Country	Gender	Age	Date of collection
MRC-DPRU861	G12P[6]	ETH	Male	8 months	Mar-2013
MRC-DPRU2130	G12P[6]	ZAF	Female	6 months	Sep-2005
MRC-DPRU4090	G12P[6]	ZAF	Male	1 day	Jul-2011
MRC-DPRU1370	G12P[6]	ZAF	Female	4 months	Aug-2004
MRC-DPRU1656	G12P[6]	ZAF	Male	6 months	Jun-2008
MRC-DPRU3491	G12P[6]	ZMB	Male	2 months	08-Sep-09
MRC-DPRU1680	G12P[6]	ZMB	Male	8 months	19-Oct-08
MRC-DPRU3507	G12P[6]	ZMB	Male	3 months	21-Nov-09
MRC-DPRU1660	G12P[6]	ZMB	Male	3 months	03-Aug-08
MRC-DPRU3713	G12P[6]	UGA	N/A	N/A	2010
MRC-DPRU4616	G12P[6]	UGA	N/A	N/A	2011
MRC-DPRU1689	G12P[6]	TGO	Male	13 months	Jun-2005
MRC-DPRU4578	G12P[6]	TGO	Male	15 months	Oct-2010
MRC-DPRU1794	G12P[6]	ZWE	Male	12 months	Jun-2009
MRC-DPRU3043	G12P[8]	CMR	Female	9 months	2009
MRC-DPRU4970	G12P[8]	ETH	Male	12 months	Sep-2010
MRC-DPRU5002	G12P[8]	ETH	Male	12 months	Sep-2010
MRC-DPRU5010	G12P[8]	ETH	Male	8 months	Oct-2010
MRC-DPRU2140	G12P[8]	ZAF	Female	10 months	Sep-2005
MRC-DPRU108	G12P[8]	ZAF	Male	8 months	Apr-2009
MRC-DPRU123	G12P[8]	ZAF	Female	14 months	May-2009
MRC-DPRU138	G12P[8]	ZAF	Male	5 months	May-2009
MRC-DPRU1148	G12P[8]	ZAF	Female	1 month	May-2009
MRC-DPRU1191	G12P[8]	ZAF	Male	8 months	Jun-2009
MRC-DPRU309	G12P[8]	ZAF	Female	7 months	Apr-2010
MRC-DPRU1554	G12P[8]	ZAF	Male	4 months	Jul-2010
MRC-DPRU71	G12P[8]	ZAF	Male	14 months	May-2012
MRC-DPRU75	G12P[8]	ZAF	Female	32 months	May-2012
MRC-DPRU76	G12P[8]	ZAF	Male	7 months	Jun-2012
MRC-DPRU5166	G12P[8]	ZAF	N/A	N/A	N/A
MRC-DPRU5144	G12P[8]	TGO	Male	4 months	Sep-2010
MRC-DPRU5171	G12P[8]	TGO	Female	6 months	Dec-2010
MRC-DPRU1858	G12P[8]	ZWE	Female	9 months	Jul-2005
MRC-DPRU850	G12P[6]	ETH	Female	9 months	Nov-2012

*N/A, data not available.*

**TABLE 2:** Genotype constellations of the 34 African G12P[6] and G12P[8] strains examined in this study.

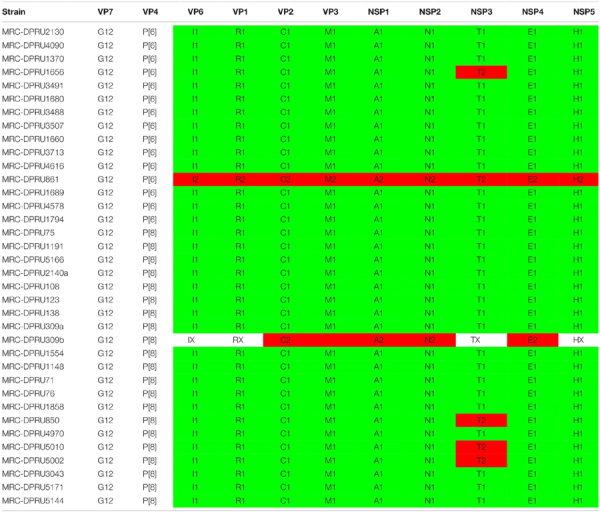

*X for unknown, The green and red background indicating the genogroup 1 (Wa-like) and genogroup 2 (DS-1) gene segments, respectively.*

In line with the majority of worldwide circulating G12 strains, the African G12P[8] strains displayed a Wa-like genetic backbone with three exceptions from Ethiopia (MRC-DPRU850, MRC-DPRU5002, and MRC-DPRU5010) and South Africa (MRC-DPRU1656), which exhibited an NSP3 of T2 mono-reassortant, within a genotype 1 constellation. Interestingly, the South African G12P[8] strain, MRC-DPRU2140, was found to contain two un-identical segments of the VP2 gene which were determined to both belong to the Wa-like genotype. The VP2 of MRC-DPRU2140a (accession number: KJ753594.1) exhibited 99.96%(100%) identity to a mixed infection South African strain, MRC-DPRU1940 which circulated in 2005 while MRC-DPRU2140b (KJ753595.1) s was homologous to study strain MRC-DPRU1858, which circulated in Zimbabwe in 2011. We have excluded further description of this strain in the manuscript. In addition, South African strain MRC-DPRU309_B contained a Wa-like back-bone with additional DS-1-like segments 2 (VP2), 5 (NSP1), 6 (NSP2), and 10 (NSP4) (Table 2), usually typical of mixed infections in case of a co-infecting strain where some of the gene segments are partially sequenced (Nyaga et al., 2015).

**FIGURE 1:**
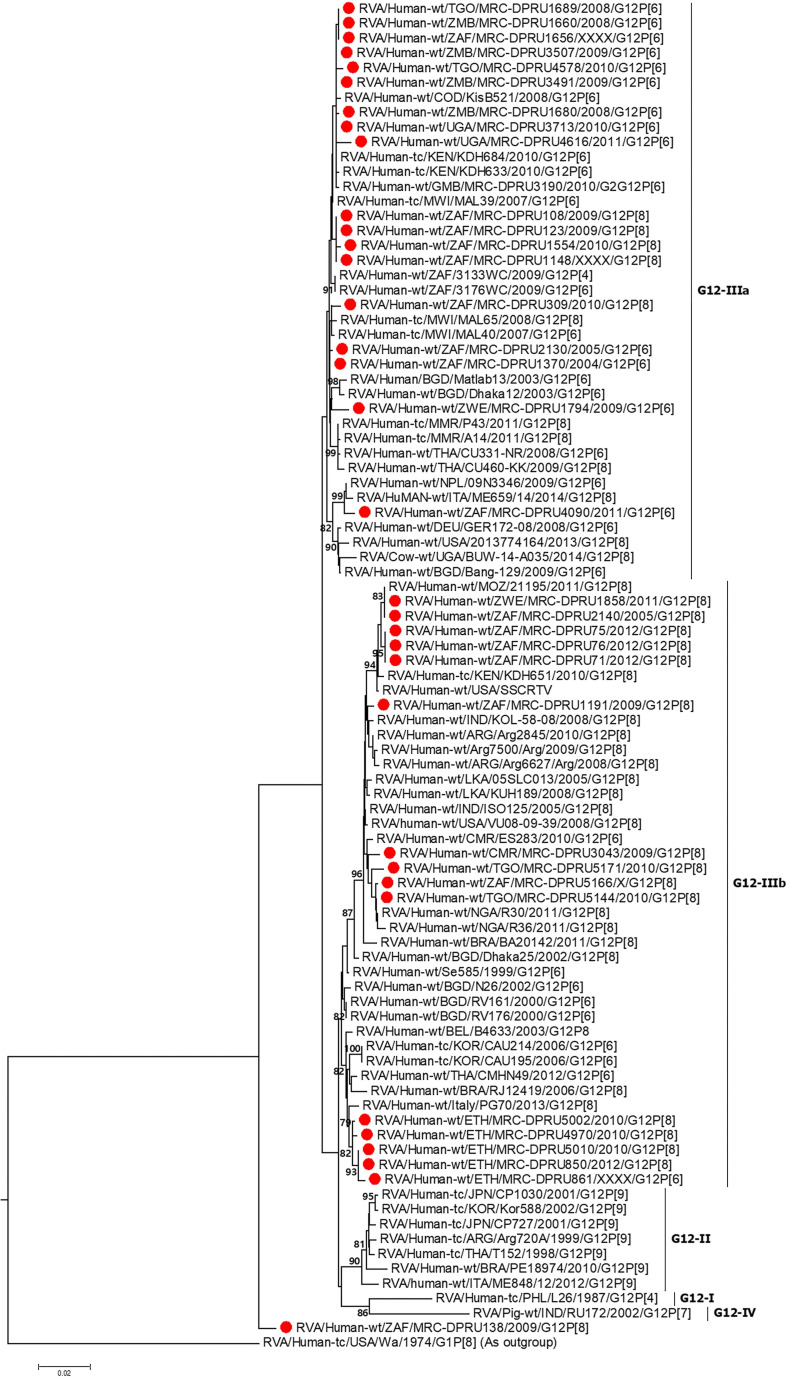
VP7 maximum likelihood tree was constructed using the general time reversible model with gamma distribution and invariant sites the tree, African G12 study strains shown in red. Bootstrap values of ≥75% are not shown. Scale bars, 0.02 substitutions per nucleotide.

Overall, the VP7 of the African G12 study strains clustered in lineage-3, together with worldwide G12P[8] and G12P[6] human rotavirus strains which were in circulation between 2000 and 2015 (Figure 1). Interestingly, the strains from Ethiopia showed distinct differences. First, the genetic backbone of the G12P[6] strain (MRC-DPRU861) belonged to the DS-1-like genogroup. Secondly, three of the four G12P[8] strains exhibited a similar mono-reassortant T2 within the NSP3, distinct from the genotype 1 in the other 8 genes of strains MRC-DPRU850, MRC-DPRU5010 and MRC-DPRU5002. Strain MRC-DPRU1656 from South Africa was the only G12P[6] found to exhibit a similar mono-reassortant T2 within the NSP3 (Table 2). The 11 segments of a one Ethiopian G12P[8] strain (MRC-DPRU4970) was phylogenetically and genetically similar to cognate gene sequences of other global Wa-like G12P[8] strains.

### Phylogenetic and Sequence Analyses

#### VP7 Analysis

A comparison of the VP7 of G12P[6] and G12P[8] study strains indicated nucleotide (amino acid) identity in the of ranges of 95.3–100% (93.3–100%) amongst themselves (data not shown). The South African G12P[6] strain (MRC-DPRU1656), was homologous to strains MRC-DPRU1689 (Togo) and MRC-DPRU1660 (Zambia) all circulating in 2008, and will be utilized as the standard reference strain for genomic comparison with globally circulating strains. The G12P[6] African study strains, displayed nucleotide (amino acid) sequence identities in the range of 99.0–99.8% (98.8–100%) and 98.7–99.5% (93.2–99.6%) in relation to strains KDH633 from Kenya and MAL39 from Malawi, and consequently formed phylogenetic clusters together with these strains (Figure 1).

MRC-DPRU1656 (G12P[6]) was moderately similar and genetically diverse nucleotide and amino acid identities of = 96.7 and 93.3%, respectively, compared to three South African G12P[8] strains (MRC-DPRU71, MRC-DPRU75 and MRC-DPRU76) which were circulating in 2012. However, these three South African strains, along with the remaining G12P[8] study strains formed clusters with cognate globally available G12P[8] strains included in this analysis (Figure 1).

#### VP4 Analysis

**FIGURE 2:**
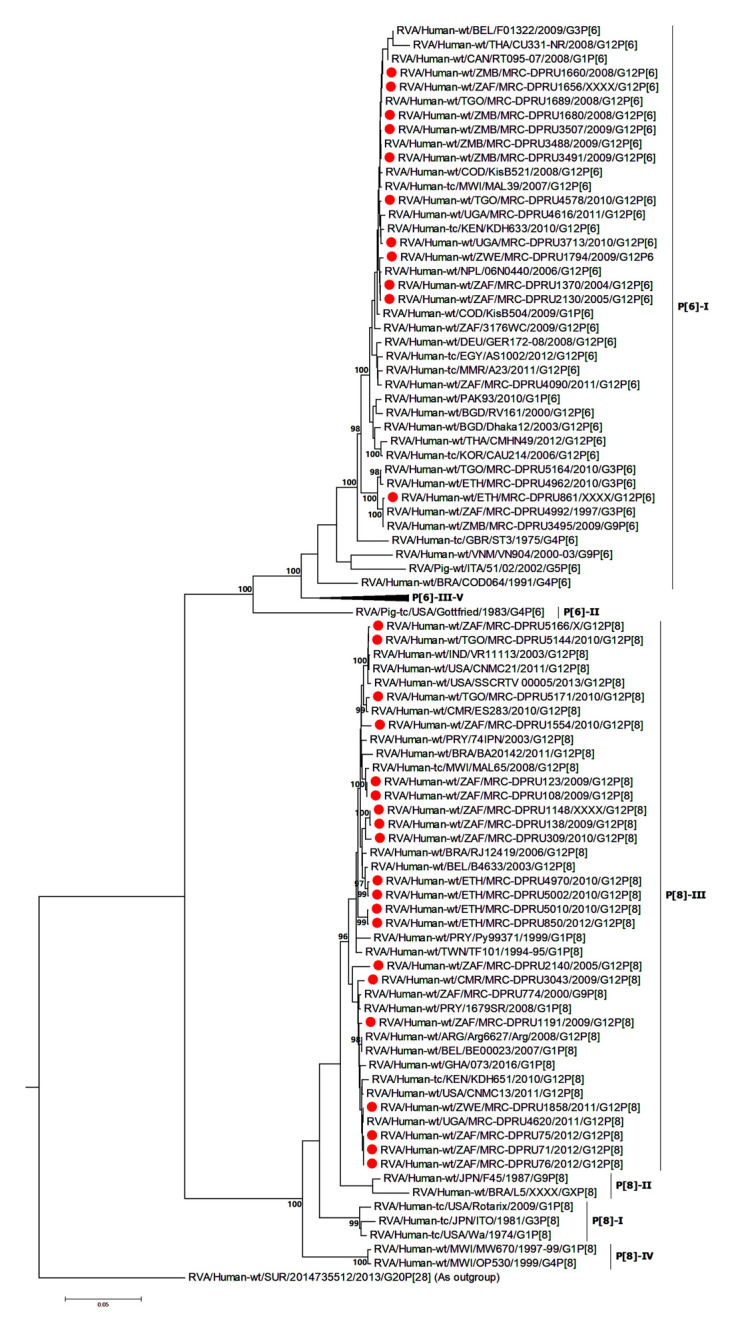
VP4 maximum likelihood tree general time reversible model with gamma distribution and invariant sites the tree. African G12 study strains shown in red. Bootstrap values of ≥75% are not shown. Scale bars, 0.05 substitutions per nucleotide.

Sequence identity among the South African G12P[6] strains, exhibited similarity in the range of 95.7–100% to each other, while the G12P[8] study strains shared 96.7–100% (94.4–100%) nucleotide (amino acid) sequence identities amongst themselves. The G12P[6] study strains MRC-DPRU3491, MRC-DPRU3488 and MRC-DPRU3507 all detected from Zambia were homologous across the genome. For the G12P[8] study strains, strains MRC-DPRU4616 from Uganda and MRC-DPRU1858 from Zimbabwe were homologous. Compared to other globally circulating G12P[6] and G12P[8] strains, the African study strains shared nucleotide (amino acid) sequence similarities in the range of 96.3–99.7% (91.6–100%) and 96.2–99.6% (97.0–99.2%), respectively. All study P[6] genotype clustered in P[6]-I and P[8] genotype in P[8]-III (Figure 2). The VP4 P[6] of Ethiopia strain MRC-DPRU861 with a DS-1-like genetic backbone clustered distinctly with other G12P[6] in lineage-I, and were closely related to strains MRC-DPRU4992 detected in South Africa in 1997, and MRC-DPRU3495 detected in Zambia in 2009 with nucleotide (amino acid) identities of 95.8% (96.2%).

#### VP1, VP2, VP3, and VP6 Analyses

Study strains belonging to genogroup 1 (Wa-like backbone) exhibited nucleotide (amino acid) sequence identities in the range of 97.2–100% (98.4–99.6%), 97.3–99.8% (91.2–99.7%), 97.7–99.9% (97.0–99.6%), and 95.4–100% (97.9–100%) amongst themselves at the VP1, VP2, VP3 and VP6 segments, respectively. Phylogenetic analysis of the VP1 (R1) and VP3 (M1) genes segments revealed a distinct segregation wherein the majority of the study strains were found in large contemporary R1 and M1 clusters, in common with cognate gene sequences of a global collection of circulating G12, G3, G4, and G9 rotaviruses (Supplementary Figures S1A,C). The South African strains (MRC-DPRU71, MRC-DPRU75, and MRC-DPRU76) clustered separately, exhibiting a close relationship to a G1P[8] strain which was detected in Malawi in 2013 (nucleotide identity = 99.6%; amino acid = 100%) In the M1 cluster, two Ethiopian strains (MRC-DPRU850 and MRC-DPRU5010) clustered with globally detected G1P[8] strains exhibiting nucleotide (amino acid) identities in the ranges of 90.1–90.7% (93.6–95.7%) to strains detected in South Africa and Belgium (Supplementary Figures S1A,C). Strain MRC-DPRU861 carrying a DS-1-like backbone, was found to share a close relationship, nucleotide (amino acid) identity 99.0% (99.6%), with the South African G3 strain, MRC-DPRU4992 which circulated in 1997 in the R2 genotype cluster (Supplementary Figure S1A), and also, exhibited a close relationship to strain 1,076 isolated from Sweden M2 genotype cluster (Supplementary Figure S1C). The MRC-DPRU309_B showed a more closer relationship to a G2P[4] strain MRC-DPRU1132 and a G2/G9P[6] mixed strain MRC-DPRU1158 from Zimbabwe (Supplementary Figure S1C).

For the VP2 gene, study strain MRC-DPRU1656 from South Africa was absolutely homologous to strain MRC-DPRU1689 from Togo. These strains, exhibited a close relationship to the Belgian G1P[8] strain BE00112 (nucleotide identity = 98.7%; amino acid identity-99.5%), in the genotype C1. The remaining study strains in genotype C1 were found to be related to cognate gene sequences of a global collection of circulating G3, G9 and G12 rotaviruses (Supplementary Figure S1B). In the genotype C2 cluster, strain MRC-DPRU861 was found in a common branch with a G2P[6] strain MRC-DPRU224, detected in Ethiopia in 2009 exhibiting a nucleotide (amino acid) identity of 99.6% (99.2%) (Supplementary Figure S1B). The MRC-DPRU309_B, clustered with mixed strains MRC-DPRU1195 and MRC-DPRU1158 detected in South Africa and Zimbabwe, respectively. Interestingly, these Africa mixed strains displayed nucleotide (amino acid) identity in the range of 96.5–96.6% (94.8–98.2%) amongst themselves.

The VP6 phylogenetic analysis of African strains in genotype I1 clustered with the VP6 gene of a global collection of G12, G9 and G1 rotaviruses (Supplementary Figure S1D). Meanwhile, strain MRC-DPRU861, in the I2 genotype was absolutely homologous to cognate gene sequence of a G3P[6] strain MRC-DPRU4962 from Ethiopia. These two strains shared a close relation to other strains from the African Rotavirus Network with the DS-1-like backbone, including G2, G8, and G12 strains (Supplementary Figure S1D).

#### NSP1–NSP5 Analysis

Amongst study strain, the non-structural genes (NSP1, NSP2, NSP3, NSP4, and NSP5) exhibited moderate to high nucleotide (amino acid) identities in the range of 95.5–99.8% (94.3–100%), 96.9–100% (97.0–100%), 95.3–99.7% (97.4–100%), 85.8–100% (86.8–100%), and 97.7–100% (97.0–100%), respectively.

Phylogenetically, NSP1 segregated into separate clusters, with the majority of the study strains clustering together with a collection of globally circulating G12 strains and shared a sequence identity in the range of 97.3–99.9%), and the Ethiopian G12P[8] strains (MRC-DPRU5002 and MRC-DPRU4970) clustered with G1P[8] strains (Supplementary Figure S2A). Not surprisingly, in the A2 genotype cluster, strain MRC-DPRU861 clustered with worldwide circulating human G2P[4] strains. The MRC-DPRU309_B on the other hand displayed a close relationship (nucleotide identity = 99.4%; amino acid identity = 99.7%) to a G2P[4] strain MRC-DPRU1132 that was detected in eSwatini. The study strains in genotype H1 cluster carrying a Wa-like genetic backbone showed the closest relationships with worldwide circulating G12, G1 and G3 strains. The MRC-DPRU861 strain exhibited a nucleotide (amino acid) identity of 98.5% (98.3%) and close relationship to two G12P[6] strains detected in Malawi (MAL39 and MAL88) in 2007 (Supplementary Figure S2B).

**FIGURE 3:**
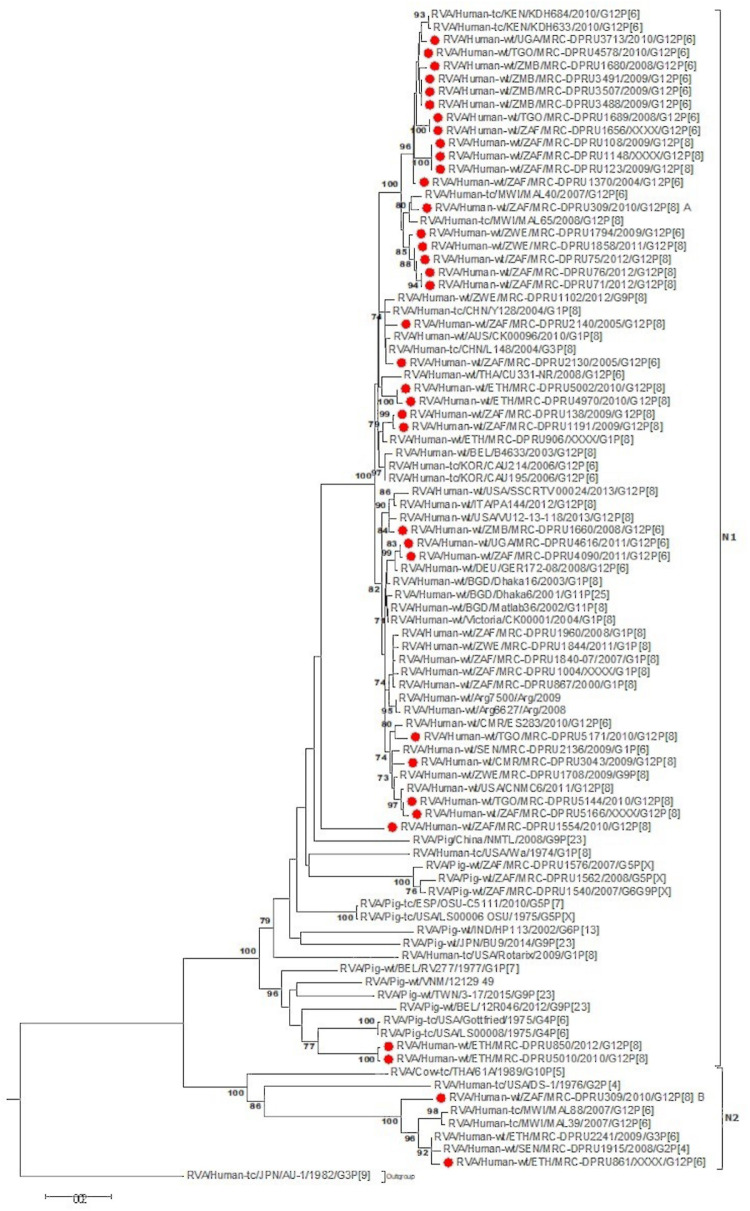
NSP2 maximum likelihood tree was constructed using the Hasegawa-Kishino-Yano model with gamma distribution and invariant sites the tree, African G12 study strains shown in red. Bootstrap values of ≥75% are not shown. Scale bars, 0.5 substitutions per nucleotide.

**FIGURE 4:**
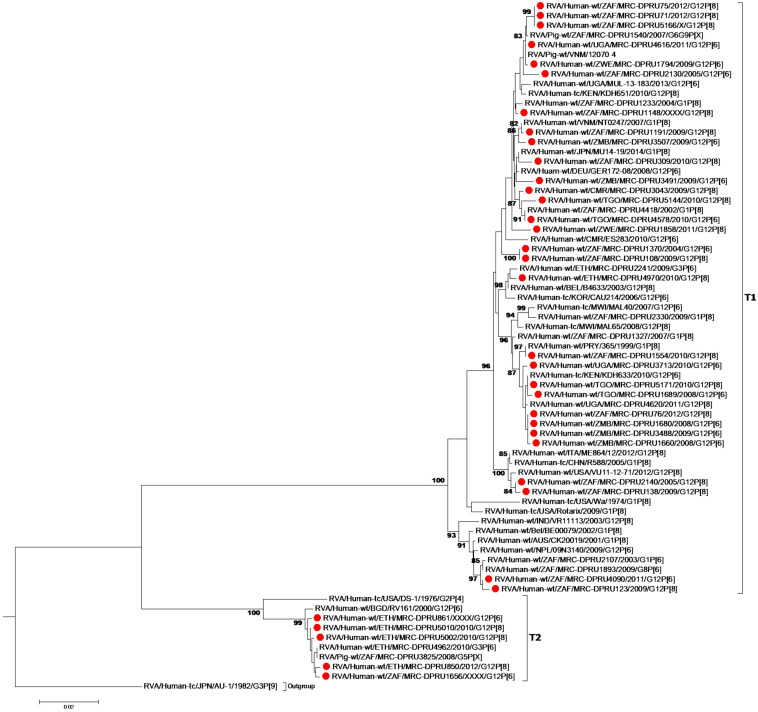
NSP3 maximum likelihood tree was constructed using the Hasegawa-Kishino-Yano model with gamma distribution and invariant sites tree, African G12 study strains shown in red. Bootstrap values of ≥75% are not shown. Scale bars, 0.02 substitutions per nucleotide.

**FIGURE 5:**
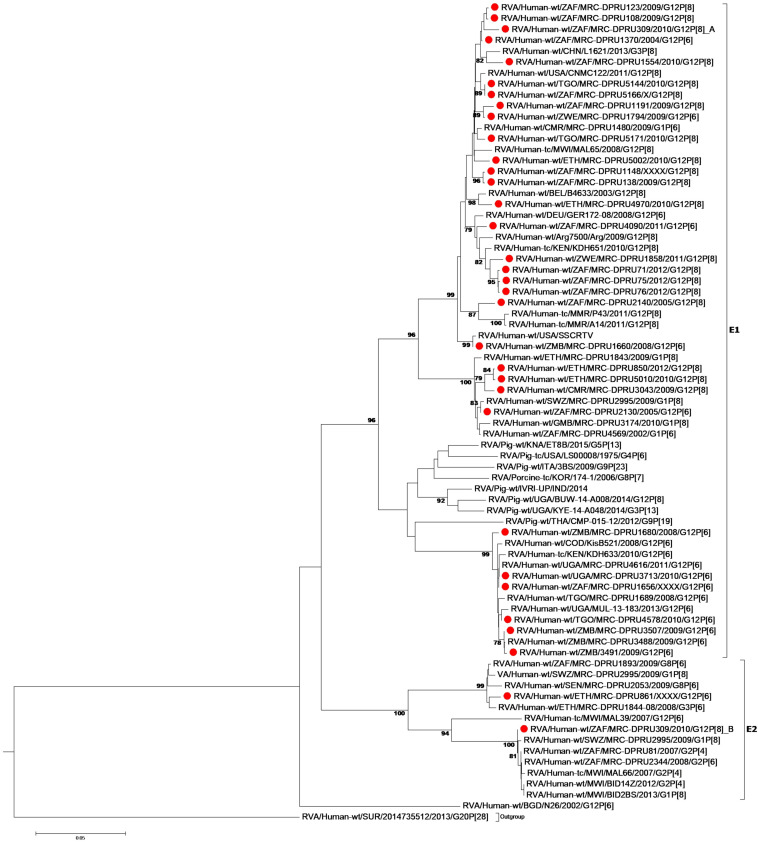
NSP4 maximum likelihood tree was constructed using the Kishino-Yano model with gamma distribution and invariant sites tree. African G12 study strains shown in red. Bootstrap values of ≥75% are not shown. Scale bars, 0.05 substitutions per nucleotide.

At the NSP2-NSP4 gene segments, the majority of the study strains belonging to N1, T1, and E1, genotypes and exhibited closest relationships, nucleotide (amino acid) identity of 98.6 (99.7%), with similar gene sequences of worldwide collection of circulating G12, G1 and G3 strains (Figures 3–5). Similarly, the Ethiopian mono-reassortant strains (MRC-DPRU 5002 and MRC-DPRU850) consistently clustered with cognate gene sequences of global G1P[8] strains at the N1, T1, and E1 genotype clusters displaying nucleotide (amino acid) similarities in the range of 98.2–98.6% (99.0–99.3%). Some human-like porcine strains (such as MRC-DPRU5140) and porcine strains (such as12070) were grouped together in the N1, T1, and E1 genotype clusters (Figures 3–5).

Strains with both P[6] and P[8] characteristics from multiple countries (e.g., G12P[6] MRC-DPRU4616 (Uganda) and MRC-DPRU2130 (South Africa); and G12P[8] strains such as the Zimbabwean MRC-DPRU1794 and the South Africa strains (MRC-DPRU71, MRC-DPRU5166, and MRC-DPRU75) shared >98% nucleotide sequence identity to porcine RVA strains in GenBank (e.g., MRC-DPRU1540 accession number: KJ753681.1 and 12070_4 accession number: KX363291.1) at the NSP3 gene segment (Figure 4). These six strains are likely to be porcine-human reassortants. Interestingly, strains MRC-DPRU850 and MRC-DPRU5010 also displayed close clustering with strain Pig-tc/USA/LS00008/1975/G4P[6], and shared a nucleotide identity of 95.9 and 95.0%, respectively.

The DS-1 like strain MRC-DPRU861 was found to be related to African G2P[4], G2P[6], and G3P[6] strains, also identified in this laboratory (Figures 3–5), the closest relationship was seen between this study strain and the unusual G3P[6] strain (MRC-DPRU4962, accession number: KJ751729.1), also detected in Ethiopia. It is possible that strain MRC-DPRU 309_B is another reassortant within the Wa-like G12P[6] strains considering the close clustering with the G12P[6] strains in genotype N2 and E2, exhibiting nucleotide (amino acid) identities = 99.1–99.3% (98.3–99.0%) to MAL39 strains collected in Malawi in 2007. In addition, four Ethiopian G12P[8] and the only G12P[6] strain from South Africa in the T2 genotype cluster, exhibited a close relationship (nucleotide (amino acid) identity in the range of 99.5–99.8% (99.0–100%) to the South African RVA porcine strain MRC-DPRU3825 (accession number: KJ753141.1) (Figure 4).

## Discussion

The increasing detection of G12 strains as predominant genotypes during the annual surveillance seasons of several countries worldwide, particularly in Africa and Asia, over the last few years, suggests a strong adaptation of these RVA to infect and spread among humans and animals. Therefore, studying the evolution of RVA G12 strains remains highly relevant in Africa and elsewhere, where G12 strains of various VP4 genotypes circulate across the continent. Previous studies which have analyzed the full genome of selected RVA G12P[6] and G12P[8] strains from Brazil (Gómez et al., 2014), Kenya (Komoto et al., 2014), Myanmar (Ide et al., 2015), Italy (De Grazia et al., 2015), Thailand (Saikruang et al., 2016), and Malawi (Nakagomi et al., 2017) have suggested that G12 strains are more frequently associated with genotype 1 constellation and rarely with genotype 2 constellation. Since a genotype 1 constellation is believed to be a stable genetic constellation of human RVA, it might have conferred an evolutionary advantage making it possible for G12 strains to adapt to the human host once introduced, and rapidly spread across the world (Matthijnssens et al., 2008; Matthijnssens and Van Ranst, 2012). Our study, evaluating the largest collection of African G12 strains with P[6] and P[8], confirms this observation, as only one G12P[6] strain, MRC-DPRU861 from Ethiopia, was shown to have acquired a pure DS-1-like genomic constellation (G12-P[6]-I2-R2-C2-M2-A2-N2-T2-E2-H2) while the remaining majority (n = 29) of the analyzed G12P[6]/[8] study strains exhibited a typical genotype 1 backbone. Four strains showed evidence of genetic reassortment of the NSP3 gene with T2 from a genotype 2 backbone.

Phylogenetic analysis of the 9-genome segments (excluding VP7 and VP4) identified a segregation of the African G12 strains examined in the study into the two genotypes, 1 or 2, based on the Wa-like or DS-1-like genetic constellations. The African G12 strains in genotype 1 Wa-like clusters were found to be genetically similar to globally circulating G12, G9, and G1 strains in large contemporary, diverse Wa-like clusters with RVA isolated from numerous countries. African study strains which were circulating in South Africa, Uganda and Zimbabwe between 2011 and 2013 appear to be a slightly later variation of the G12 strains than the majority in this study which were circulating between 2004 and 2012.

Furthermore, South African G12 strains isolated from 2004 to 2012 which constituted the majority of the study strains were most likely derived from distinct origins. The G12P[6] strains (MRC-DPRU1370 and MRC-DPRU2130) which were isolated in 2004 and 2005, respectively, were found to be closely related to strains 3133WC (South Africa), one of the first G12 strains to be characterized by full genome sequencing in Africa (Jere et al., 2011), and GER172-08 (Germany) which was isolated from a newborn in 2008 (Pietsch and Liebert, 2009). Strains 3133WC and GER172-08 showed close relationships to Southeastern Asian and Belgian G12 strains, indicating global circulation. Another South African G12P[6] strain isolated in 2008 (MRC-DPRU1656) displayed significant homology to G12P[6] strains circulating in Togo (MRC-DPRU1689) and Uganda (MRC-DPRU3713) the same year, and exhibited the closest relationship to the G12P[6] strains from Kenya (KHD633) circulating in 2010 (Komoto et al., 2014), showing the continental-wide spread of these strains over a 10-year period.

The G12P[8] MRC-DPRU1191, collected from a South African infant in 2009 clustered separately and with highly identical strains South African strains that same year (MRC-DPRU108, MRC-DPRU123, and MRC-DPRU138) and all exhibited a close relationship to G12 strains which circulated in Malawi in 2007 (Nakagomi et al., 2017), Democratic Republic of the Congo in 2008 (Heylen et al., 2014) and several other regions globally including the United States and Latin America, India and Myanmar (see Figure 1), all circulating between 2005 and 2011. Thus, a global circulation of related G12P[6] and G12P[8] strains over the same time period can be seen.

The identical G12P[8] strains, MRC-DPRU71, MRC-DPRU75, MRC-DPRU76, and MRC-DPRU1148 which were circulating in South Africa in 2012, were found in a common cluster with study G12P[8] strains MRC-DPRU4620 (Uganda), MRC-DPRU1858 (Zimbabwe) and MRC-DPRU1660 (Zambia) and shared the highest genetic relationship to G12 strains KDH651 (Kenya), CU33-NR (Thailand) and VU-08-09-39 (United States), circulating between 2008 and 2010. Significantly, it has been shown that although the German and African G12 RVA strains carried a Wa-like backbone, these strains were likely derived from separate reassortment events with other co-circulating RVA genotype 1 strains G1P[8] and G9P[8] (Pietsch and Liebert, 2009; Komoto et al., 2014).

Similarly, of the four G12P[8] strains detected in Ethiopia in 2010, two were closely related to each other at all 10 gene segments indicating the derivation of these two strains from a common origin. The outstanding gene was NSP3 which in one strain was genotype 1 at T1 (MRC-DPRU4970) and the other was genotype 2 at T2 (MRC-DPRU5002, representing the mono-reassortants described earlier). The other two mono-reassortants (MRC-DPRU5010 and MRC-DPRU850) on the other hand, appeared to be of a more distinct origin sharing a close relationship and circulating 2 years later. These tended to cluster closely to G1P[8] strains from South Africa and Belgium. Overall, these results indicate interaction and evidence for frequent occurrence of genetic reassortment between globally circulating G12P[8], G12P[6], G12P[4], G1P[8], and G9P[8] and the African G12 strains. Our observations support the hypothesis of co-existence of different genetic clones during the spread of G12P[6] and G12P[8] strains in Africa, possibly generated by multiple reassortment events (Pietsch and Liebert, 2009; Ndze et al., 2013a,b; Gómez et al., 2014; Komoto et al., 2014; Wangchuk et al., 2014; Delogu et al., 2015; Ide et al., 2015).

Due to the segmented nature of their genome, rotavirus genetic and phenotypic characteristics change over time. The viral genome sequence variation occurs by either nucleotide substitutions, recombination events or reassortment of individual genes (Doro et al., 2014). Reassortant G12 strains showing complex gene combinations have been sporadically reported (Komoto et al., 2014; Bwogi et al., 2017; Nakagomi et al., 2017). Such strains have been considered to be the product of multiple reassortment steps involving both human and animal strains (Wakuda et al., 2003). Our study reports intergroup and interspecies reassortment. Three G12P[8] strains (MRC-DPRU850, MRC-DPRU5002, and MRC-DPRU5010) detected from Ethiopia acquired DS-1-like genotype (T2) at the NSP3 gene. These strains are likely to have originally carried a typical Wa-like genotype constellation and a single reassortment event occurred which resulted in strains that could circulate for at least 2 years in the population. Direct reassortment with porcine or porcine-like human RVA, such as those reported in Kenya (Komoto et al., 2014) and DRC (Heylen et al., 2014) potentially led to the emergence of NSP2, NSP3 and NSP4 genotype reassortment events in strains in nature. Since porcine rotavirus reassortment with G12 strains have not been seen elsewhere in the world, it is plausible that strains carrying this porcine and human-porcine like genes evolved in Africa.

We speculate that the DS-1-like Ethiopian G12P[6] strain (MRC-DPRU861) may have occurred as a result of multiple reassortant events between an African Wa-like G12P[6] strain with G3P[6] and G2P[4] strains. We previously reported a G2P[4] strain (MRC-DPRU1915, accession number: KJ753528.1) from Senegal circulating in 2008 which displayed absolute amino acid and 99.5% nucleotide identify at the NSP1 and NSP2 genes to these two study strains. The remaining segments were more likely originated from the G3P[6] strains detected in Ethiopia (accession number: KJ751729.1).

Genome reassortment and gene mutation are likely to be the key contributing factors to rapid spread of G12P[6] and G12P[8] strains that circulated in Africa between 2004 and 2012. Our genomic characterization resulted in the identification of porcine reassortant NSP2, NSP3 and NSP4 genes, an adaptation which has evolved in Africa. The results underscore the importance of complete genome characterization of RVA strains and the need for continued surveillance of RVA globally.

## Data Availability Statement

The datasets generated for this study can be found in the online repositories. The names of the repository/repositories and accession number(s) can be found in the article/Supplementary Material.

## Author Contributions

FM, ME, LS, MN, JM, MM, and AS conceived and designed the experiments. NM, ArM, AuM, JS, AA, AB, ET, KR, and IP performed the experiments. FM, ME, MN, JM, and AS analyzed the data and wrote the manuscript with contributions from all other authors. All authors contributed to the article and approved the submitted version.

## Disclaimer

The findings and conclusions of the study are those of the authors and do not necessarily reflect the position of the World Health Organization, or the Bill & Melinda Gates Foundation, or the US CDC.

## Conflict of Interest

AS was employed by the Bill & Melinda Gates Foundation which is working on rotavirus vaccine development and deployment. Understanding rotavirus strain diversity and evolution, including under possible vaccine pressure, is an important goal. The Bill & Melinda Gates Foundation partially funded the African Enteric Viruses Genome Initiative (AEVGI) at the University of Free State, but was not involved in sample selection or the laboratory methodology used. AS was engaged in the interpretation of the data and the drafting and approval of the manuscript as the senior corresponding author. The remaining authors declare that the research was conducted in the absence of any commercial or financial relationships that could be construed as a potential conflict of interest.

## Footnotes

1 https://rega.kuleuven.be/cev/viralmetagenomics/virus-classification

## Supplementary Material

Supplementary Figure 1 Maximum likelihood trees for VP1 **(A)**, VP2 **(B)**, VP3 **(C)**, and VP6 **(D)** constructed using Hasegawa-Kishino-Yano model with gamma distribution and invariant sites for the VP1, VP2, and VP3 tree and T92 with gamma distribution and invariant sites for the VP6 tree.

Supplementary Figure 2 NSP1 and NSP5 maximum likelihood tree was constructed using the general time reversible model with gamma distribution and invariant sites. African G12 study strains shown in red. Bootstrap values of ≥75% are not shown. Scale bars, 0.02 substitutions per nucleotide.
