# Supplementary figures and images for: Whole Genome Analysis of African G12P[6] and G12P[8] Rotaviruses Provides Evidence of Porcine-Human Reassortment at NSP2, NSP3, and NSP4
Source: Front Microbiol. 2021 Jan 12;11:604444. doi: 10.3389/fmicb.2020.604444 (PMC7835662; doi:10.3389/fmicb.2020.604444)

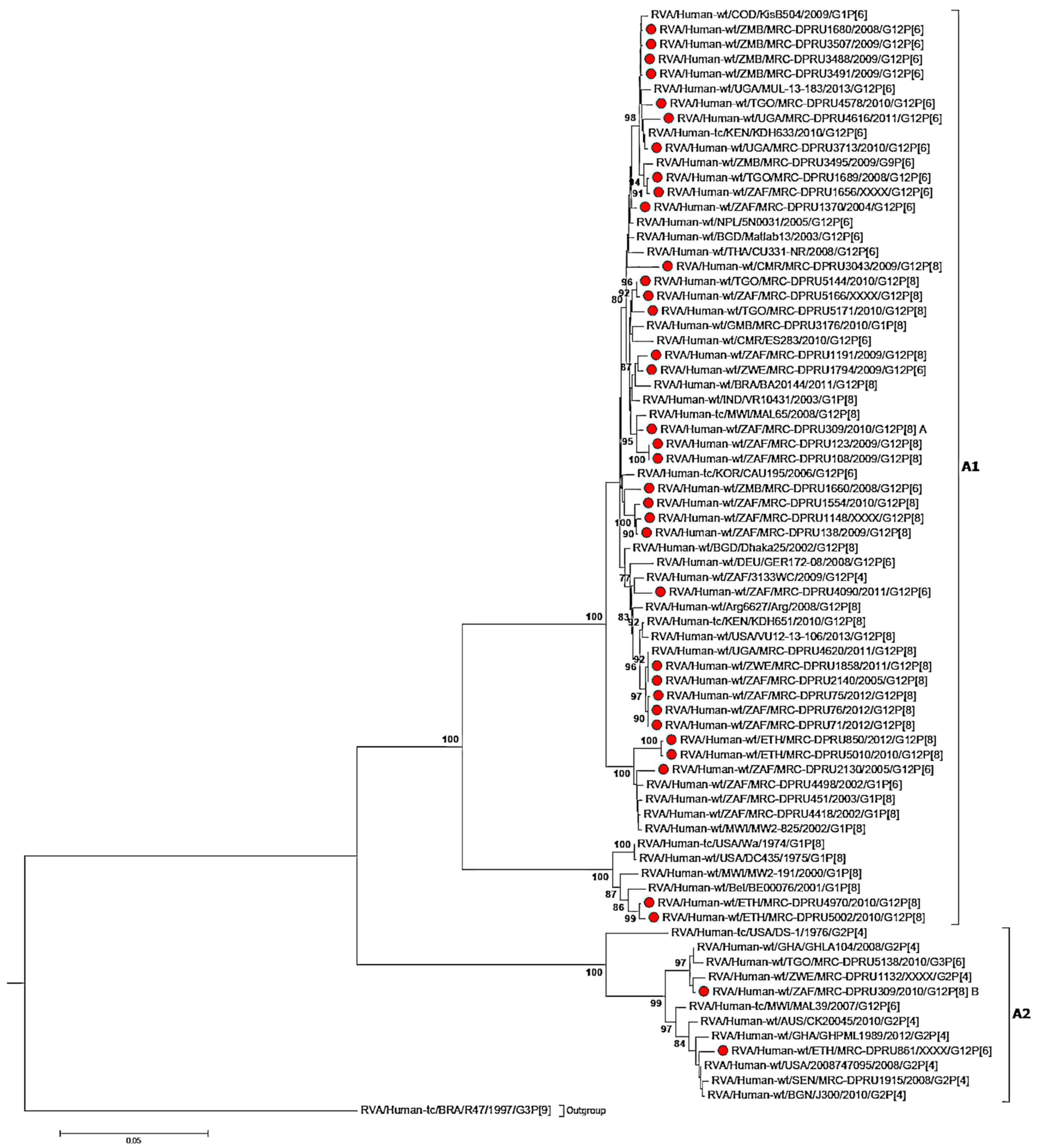

Supplement: Supplementary Figure 2 — NSP1 and NSP5 maximum likelihood tree was constructed using the general time reversible model with gamma distribution and invariant sites. African G12 study strains shown in red. Bootstrap values of ≥75% are not shown. Scale bars, 0.02 substitutions per nucleotide. [file Image_2.TIF]

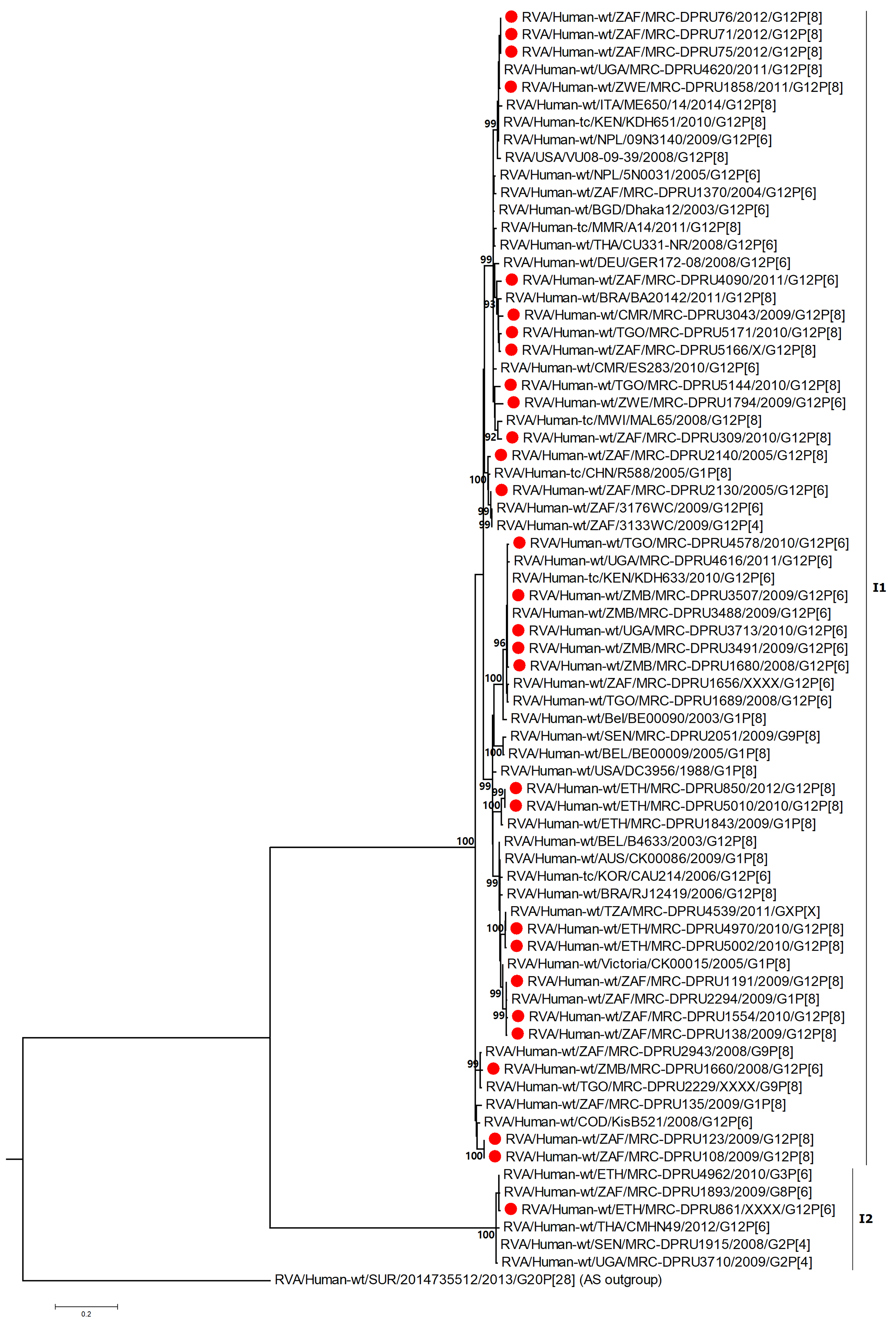

Supplement: Supplementary file 3 [file Image_3.JPEG]

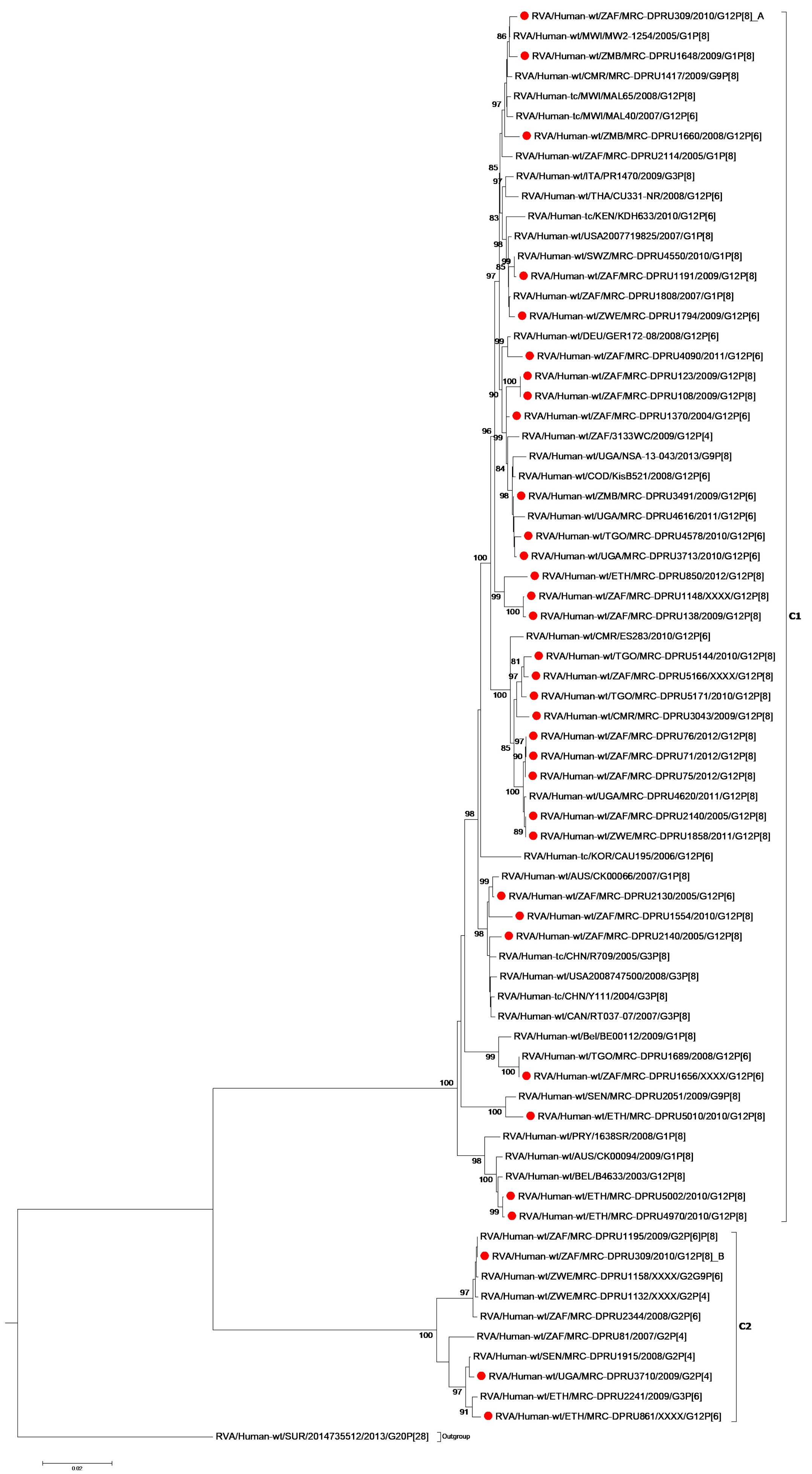

Supplement: Supplementary file 4 [file Image_4.TIF]

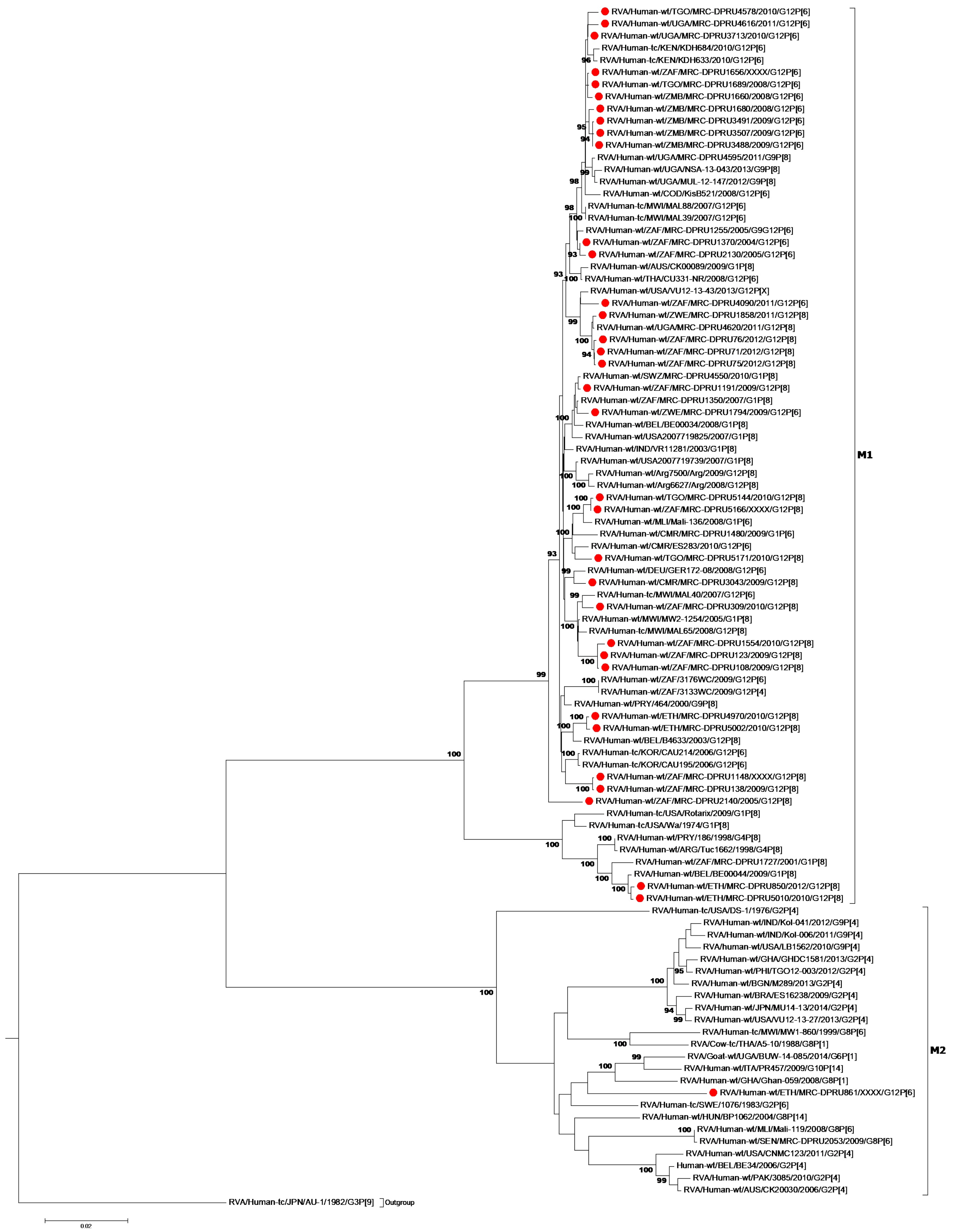

Supplement: Supplementary file 5 [file Image_5.TIF]

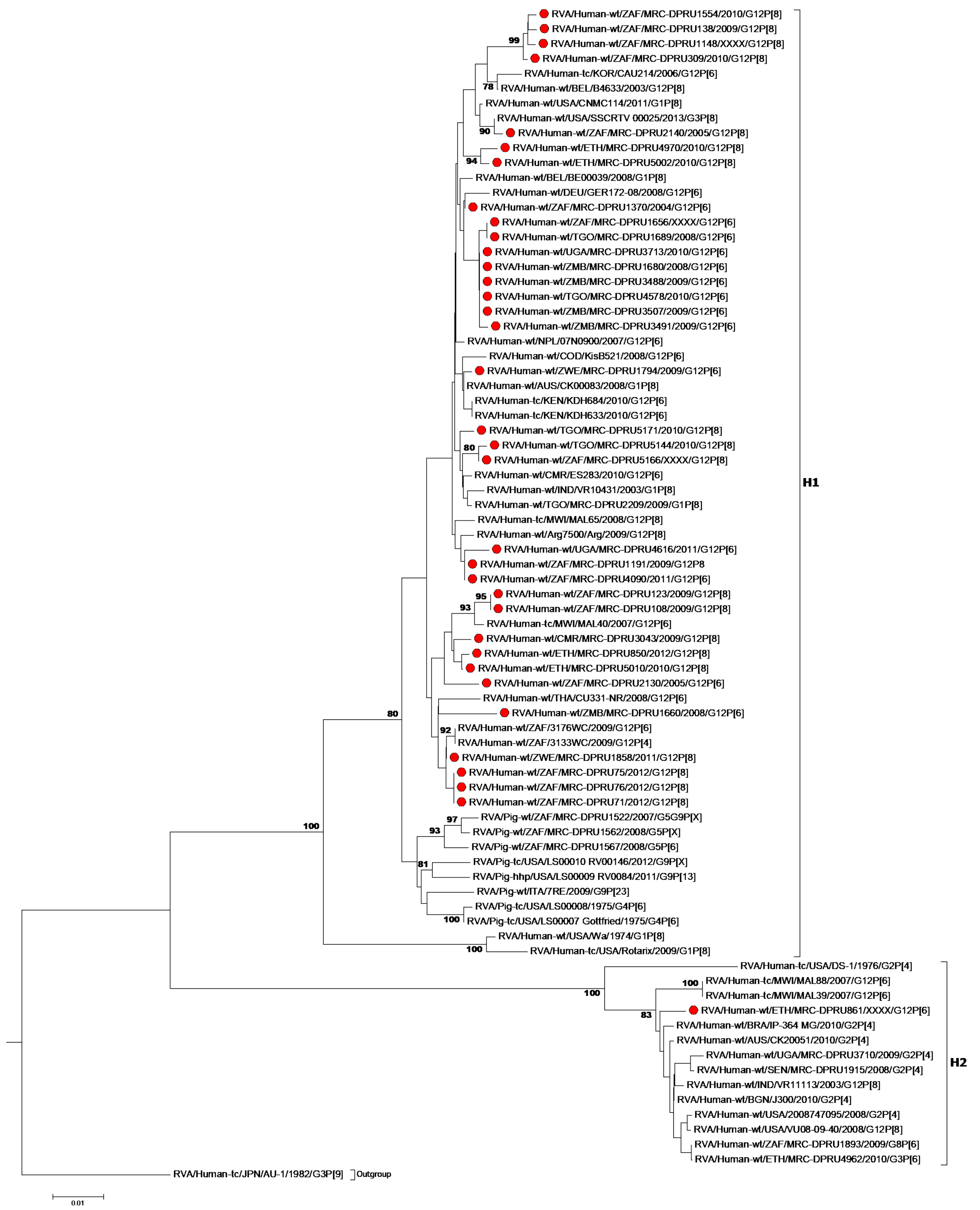

Supplement: Supplementary file 6 [file Image_6.TIF]
